# Supplementary material for: How prolonged expression of Hunchback, a temporal transcription factor, re-wires locomotor circuits
Source: eLife. 2019 Sep 10;8:e46089. doi: 10.7554/eLife.46089 (PMC6754208; doi:10.7554/eLife.46089)
Supplement: Figure 2—source data 1. [file elife-46089-fig2-data1.docx]

Source Data for Figure 2 G, H, I

| Genotype | Marker | Number of values | Mean (number of cells) | Std. Deviation | Std. Error of Mean | p value |
| --- | --- | --- | --- | --- | --- | --- |
| Control | GFP+ | 17 | 39.94 | 3.544 | 0.8595 | NA |
| NB7-1>Hb | GFP+ | 20 | 41.70 | 3.948 | 0.8829 | 0.166* |
|  |  |  |  |  |  |  |
| Control | Eve+ GFP+ | 100 | 5.000 | 0.000 | 0.000 | NA |
| Control | Eve+ GFP+ | 148 | 9.959 | 2.705 | 0.2224 | <0.0001** |
|  |  |  |  |  |  |  |
| Control | Cas+ Eve- GFP+ | 23 | 13.43 | 2.212 | 0.4612 | NA |
| NB7-1>Hb | Cas+ Eve- GFP+ | 30 | 0.2667 | 0.5833 | 0.1065 | <0.0001** |

*Unpaired t test, two-tailed

**Unpaired t test, two-tailed with Welch’s correction (for un-equal Std. Deviation)
