# Supplementary material for: How prolonged expression of Hunchback, a temporal transcription factor, re-wires locomotor circuits
Source: eLife. 2019 Sep 10;8:e46089. doi: 10.7554/eLife.46089 (PMC6754208; doi:10.7554/eLife.46089)
Supplement: Figure 5—source data 1. [file elife-46089-fig5-data1.docx]

Source Data for Figure 5I-K

|  | Genotype | Number of values | Mean  (1b branch number) | Std. Deviation | Std. Error of Mean | p value |
| --- | --- | --- | --- | --- | --- | --- |
| muscle 9 | UAS-Hb/+ | 32 | 2.406 | 0.6148 | 0.1087 | NA |
|  | U MN>Hb | 27 | 2,296 | 0.6688 | 0.1287 | 0.9982 |
|  | NB7-1>Hb | 39 | 4.538 | 2.024 | 0.3241 | <0.0001** |
|  |  |  |  |  |  |  |
| muscle 10 | UAS-Hb/+ | 35 | 2.2 | 0.901 | 0.1523 | NA |
|  | U MN>Hb | 27 | 1.815 | 0.7357 | 0.7357 | 0.9185 |
|  | NB7-1>Hb | 46 | 6.696 | 2.555 | 0.3767 | <0.0001** |
|  |  |  |  |  |  |  |
| muscle 2 | UAS-Hb/+ | 33 | 1.758 | 0.6629 | 0.1154 | NA |
|  | U MN>Hb | 27 | 2.185 | 0.7357 | 0.1416 | 0.7269 |
|  | NB7-1>Hb | 44 | 3.455 | 2.454 | 0.3699 | <0.0001** |
|  |  |  |  |  |  |  |
| muscle 4 | UAS-Hb/+ | 36 | 2.556 | 0.9085 | 0.1514 | NA |
|  | U MN>Hb | 30 | 2.433 | 0.8584 | 0.1567 | 0.9837 |
|  | NB7-1>Hb | 18 | 1.056 | 1.211 | 0.2855 | <0.0001** |
|  |  |  |  |  |  |  |
| muscle 1 | UAS-Hb/+ | 36 | 1.889 | 0.5746 | 0.09577 | NA |
|  | U MN>Hb | 27 | 2.037 | 0.7061 | 0.1359 | 0.9996* |
|  | NB7-1>Hb | 46 | 1.826 | 1.253 | 0.1847 | 0.9773* |

Source Data for Figure 5N

|  | Genotype | Number of values | Mean  (microns) | Std. Deviation | Std. Error of Mean | p value |
| --- | --- | --- | --- | --- | --- | --- |
| HRP | Control | 8 | 1.625 | 0.4175 | 0.1830 | NA |
|  | NB7-1>HB | 13 | 1.923 | 0,7596 | 0.2197 | n.s.*** |

*Ordinary one-way ANOVA with Dunnett correction for multiple comparison

**Brown-Forxythe and Welch ANOVA (for un-equal Std. Deviation) with Dunnett correction for multiple comparision

***t-test, with Welch’s correction (for un-equal Std. Deviation)
