# Supplementary material for: How prolonged expression of Hunchback, a temporal transcription factor, re-wires locomotor circuits
Source: eLife. 2019 Sep 10;8:e46089. doi: 10.7554/eLife.46089 (PMC6754208; doi:10.7554/eLife.46089)
Supplement: Figure 8—source data 1. [file elife-46089-fig8-data1.docx]

Source data for Figure 8C.

| Genotype | Number of values | Mean (number of cells) | Std.  Deviation | Std Error of Mean | P-value |
| --- | --- | --- | --- | --- | --- |
| Em-Control | 38 | 2.000 | 0.0000 | 0.0000 | NA |
| L3-Control | 28 | 2.000 | 0.0000 | 0.0000 | >0.9999 |
|  |  |  |  |  |  |
| Em-NB7-1>Hb | 38 | 6.605 | 2.477 | 0.4019 | NA |
| L3-NB7-1>Hb | 84 | 2.060 | 0.2380 | 0.02597 | <0.0001 |

Ordinary one-way ANOVA

Source data for Figure 8G.

| Genotype | Marker | Number of values | Mean (number of cells) | Std.  Deviation | Std Error of Mean | P-value |
| --- | --- | --- | --- | --- | --- | --- |
| Control | Hb(+) Eve(+) | 28 | 2.000 | 0.000 | 0.000 | NA |
| NB7-1>1XHb | Hb(+) Eve(+) | 68 | 2.162 | 0.3710 | 0.04499 | <0.0001 |
| NB7-1>2XHb | Hb(+) Eve(+) | 84 | 2.060 | 0.2380 | 0.02597 | <0.0001 |
| NB7-1>2XHb 29C | Hb(+) Eve(+) | 27 | 2.259 | 0.4466 | 0.08594 | <0.0001 |
| NB7-1>4XHb | Hb(+) Eve(+) | 12 | 2.083 | 0.2887 | 0.08333 | <0.0001 |
|  |  |  |  |  |  |  |
| Control | Eve(+) | 28 | 5.000 | 0.000 | 0.000 | NA |
| NB7-1>1XHb | Eve(+) | 68 | 10.79 | 4.024 | 0.4880 | <0.0001 |
| NB7-1>2XHb | Eve(+) | 84 | 12.33 | 3.500 | 0.3819 | <0.0001 |
| NB7-1>2XHb 29C | Eve(+) | 27 | 11.74 | 2.669 | 0.5136 | <0.0001 |
| NB7-1>4XHb | Eve(+) | 12 | 16.08 | 3.288 | 0.9491 | <0.0001 |

Ordinary one-way ANOVA

Source data for Figure 8H.

| Genotype | Number of values | Mean (number of branches) | Std.  Deviation | Std Error of Mean | P-value |
| --- | --- | --- | --- | --- | --- |
| 2 Hb(+) MN9-1b | 98 | 3.357 | 1.487 | 0.1502 | NA |
| 3 Hb(+) MN9-1b | 16 | 4.750 | 1.612 | 0.4031 | 0.0021 |
|  |  |  |  |  |  |
| 2 Hb(+) MN10-1b | 98 | 5.990 | 1.897 | 0.1916 | NA |
| 3 Hb(+) MN10-1b | 16 | 5.938 | 1.389 | 0.3472 | 0.3779 |

Ordinary one-way ANOVA
