# Supplementary material for: How prolonged expression of Hunchback, a temporal transcription factor, re-wires locomotor circuits
Source: eLife. 2019 Sep 10;8:e46089. doi: 10.7554/eLife.46089 (PMC6754208; doi:10.7554/eLife.46089)
Supplement: Figure 9—source data 1. [file elife-46089-fig9-data1.docx]

Figure 9-source data 1

Source data for Figure 9F.

Find in revision file>Eve Hb L3 data Fin>Data 9>need to do descriptive stats on Data 11

| Genotype | Marker | Number of values | Mean (number of cells) | Std.  Deviation | P-value |
| --- | --- | --- | --- | --- | --- |
| Control | Hb(+) Eve (+) | 28 | 2.000 | 0.000 | NA |
| VP16::Hb/+ | Hb(+) Eve (+) | 29 | 2.000 | 0.000 | >0.9999 |
| NB7-1>VP16::Hb | Hb(+) Eve (+) | 70 | 3.962 | 1.362 | <0.0001 |
| En>Svp | Hb(+) Eve (+) | 65 | 1.814 | 0.392 | 0.6146 |
|  |  |  |  |  |  |
| Control | Eve (+) | 28 | 5.000 | 0.000 | NA |
| VP16::Hb/+ | Eve (+) | 42 | 5.000 | 0.000 | >0.9999 |
| NB7-1>VP16::Hb | Eve (+) | 78 | 5.141 | 0.908 | 0.5923 |
| En>Svp | Eve (+) | 70 | 4.386 | 0.666 | 0.0001 |

Ordinary one-way ANOVA

Source data for Figure 9G.

| Number of Hb(+) Eve (+) / Genotype | Number of values | Mean (number of branches) | Std.  Deviation | P-value |
| --- | --- | --- | --- | --- |
| 1 Hb(+) En>Svp | 12 | 0.500 | 0.7977 | 0.0123 |
| 2 Hb(+) En>Svp | 44 | 2.091 | 0.6030 | 0.9997 |
| 2 C | 16 | 2.188 | 0.6551 | NA |
| 2 Hb(+) NB7-1>VP16::Hb | 14 | 2.714 | 1.326 | 0.9478 |
| 3 Hb(+) NB7-1>VP16::Hb | 16 | 3.625 | 1.893 | 0.0278 |
| 4 Hb(+) NB7-1>VP16::Hb | 21 | 3.286 | 1.848 | 0.1241 |
| 5 Hb(+) NB7-1>VP16::Hb | 14 | 4.643 | 2.951 | <0.0001 |
| 6 Hb(+) NB7-1>VP16::Hb | 13 | 5.462 | 2.787 | <0.0001 |

Ordinary one-way ANOVA

Source data for Figure 9H.

| Number of Hb(+) Eve (+) / Genotype | Number of values | Mean (number of branches) | Std.  Deviation | P-value |
| --- | --- | --- | --- | --- |
| 1 Hb(+) En>Svp | 12 | 2.250 | 0.6216 | 0.995 |
| 2 Hb(+) En>Svp | 43 | 2.326 | 0.6804 | 0.9989 |
| 2 C | 17 | 2.059 | 0.5557 | NA |
| 2 Hb(+) NB7-1>VP16::Hb | 14 | 2.571 | 0.7559 | 0.9563 |
| 3 Hb(+) NB7-1>VP16::Hb | 15 | 2.667 | 1.175 | 0.8579 |
| 4 Hb(+) NB7-1>VP16::Hb | 21 | 2.762 | 1.091 | 0.6184 |
| 5 Hb(+) NB7-1>VP16::Hb | 13 | 2.154 | 1.281 | 0.9997 |
| 6 Hb(+) NB7-1>VP16::Hb | 13 | 2.231 | 1.363 | 0.8885 |

Ordinary one-way ANOVA
